# Health Effects of Exposure to Indoor Semi-Volatile Organic Compounds in Chinese Building Environment: A Systematic Review

**DOI:** 10.3390/ijerph20010678

**Published:** 2022-12-30

**Authors:** Yeganeh Ataei, Yuexia Sun, Wei Liu, Agnes S. Ellie, Hui Dong, Umme Marium Ahmad

**Affiliations:** 1 Tianjin Key Laboratory of Indoor Air Environmental Quality Control, School of Environmental Science and Engineering, Tianjin University, Tianjin 300350, China; 2 Centre for Environmental Policy, Imperial College London, South Kensington, London SW7 2AZ, UK

**Keywords:** semi-volatile organic compounds (SVOCs), phthalate esters (PAEs), polycyclic aromatic (PAHs), health effects, building environment, China

## Abstract

People spend a considerable portion of their lives indoors; thus, the quality of the indoor environment is crucial. Semi-volatile organic compounds (SVOCs) are among the primary indoor pollutants responsible for various health risks. This paper systematically reviews the impact of SVOC exposure on human health in Chinese built environments. Based on a set of criteria, we judged 12 publications as providing sufficient information on both SVOC exposure and health effects to inform the relationship. Out of six studies on polycyclic aromatic hydrocarbons (PAHs), three observed a positive association between PAH exposure and lung cancer. Out of six studies of phthalate exposure, two studies reported a significant positive association between DEP and DiBP and asthma, between DEP and DEHP and dry cough among children, and between DBP and rhinitis among younger adults. The results of this review suggest that there might be a link between phthalate exposure and asthma and allergies, as well as a link between PAH exposure and lung cancer. However, due to the limited number of studies conducted, more evidence is necessary to definitively guide the establishment of standards for SVOC control in China.

## 1. Introduction

People’s lives are affected by the indoor air quality, or IAQ, described as the air quality inside constructions and structures. IAQ is essential to provide healthy and comfortable conditions for residents in buildings [1,2]. Around 90% of people’s time is spent inside buildings in today’s modern societies [3]. Therefore, an increased attention to safety is observed in residential places [2,4,5,6,7]. Indoor air pollution can be considered a significant source of risk for some residents because of insufficient ventilation, along with low-quality construction materials, diffusing toxic or dangerous gases together with dust [8,9].

Thus, issues associated with human health make the environmental conditions of buildings extremely important [4]. One type of indoor pollutant associated with considerable health concerns is the group of semi-volatile organic compounds (SVOCs) [10,11], classified by the World Health Organization (WHO, Geneva, Switzerland) as indoor organic substances with boiling points ranging from 240/260 to 380/400 °C [12]. Different pathways contribute to the entrance of these pollutants into buildings, among which infiltrating outdoor air, indoor combustion, spray products, as well as material additives, can be mentioned. Due to the low vapor pressures of SVOCs at a temperature of 25 °C (77 °F) (10^−9^ to 10 Pa) in comparison with volatile organic compounds (VOCs) (10 to 104 Pa), the gas or condensed phases of these compounds can typically be observed, leading to their redistribution from the primary source to the buildings’ interior and surfaces inside them, such as airborne particles, dust, and skin [11].

Reports have shown polycyclic aromatic hydrocarbons (PAHs), phthalate esters (PAEs), polychlorinated biphenyls (PCBs), and polybrominated diphenyl ethers (PBDEs) in different studies as the main SVOC chemical classes found indoors [10,13,14,15,16,17,18,19,20,21]. When organic compounds are combusted or pyrolyzed incompletely, polycyclic aromatic hydrocarbons (PAHs) will be produced as pervasive environmental pollutants [22,23], resulting in public health concerns across the globe [24,25]. Environmental tobacco smoke (ETS) is recognized as the main source of PAHs in the indoor environment, with considerable emission factors related to smoking in industrialized countries. [26]. Heating and cooking with solid fuels, namely dung, agricultural remnants, wood, or coal, particularly in flueless or unvented stoves, is considered to be the main source of indoor air pollution in developing countries [27]. More than 70 percent of homes in China utilize solid fuels for cooking and heating [28].

According to the WHO, 2.7% of the annual global burden of disease is due to household air pollution that results from fuel combustion, one of the top ten global threats to public health [29,30]. Studies show that health risks due to exposure to byproducts of solid fuel combustion are of particular concern in developing countries, with an increased risk of lung cancer [31,32,33], particularly in Chinese and Indian women who are mostly nonsmokers [32,34,35]. Among households in developing countries, the concentrations of PAHs are of particular concern.

Polybrominated diphenyl ethers (PBDEs) are additive flame retardants frequently employed in a wide range of consumer goods, including textiles, automobiles, furniture, computers, and other electronic devices [36]. The environment and individuals are exposed to PBDEs as they dissipate from the items in which they are used [37]. PBDEs in placental tissue, cord blood, and human breast milk have been found [38], substances in which they have a tendency to bioaccumulate [39] and through which they even convey to the fetus [40,41]. Li and Fu observed that the main areas polluted with PBDEs in Beijing (China) were households and electronics shops [42]. The potential of PBDEs for long-range transport, bioaccumulation, endocrine disruption, and carcinogenic and mutagenic effects [42,43] has made it of particular concern for decades.

Polychlorinated biphenyls (PCBs) are typically added to capacitors and transformers as heat-transfer and insulation fluids [44]. PCBs still pose a problem since they are frequently found in the environment, even though China is not their primary source and has had restrictions on them for decades [45]. PCBs affect an organism’s functions, including thyroid hormone levels, the immune system, and even cancer development [44]. In a Chinese cohort, it is significant to note that birth weight had an inverse relationship with placental PCB concentration; this relationship may be affected by placental disturbance [41].

Phthalates have become a constant in the modern lifestyle as developed countries begin to witness lifestyle changes in the form of new personal care products, cleaning products, furniture, building materials, paint, and more. All of these contain phthalates and are constantly present in our indoor environment. Humans are exposed to these materials through inhalation, ingestion, dermal absorption, or contact with medical tools [34,35,36]. This perpetual exposure to phthalates may be responsible for the increased numbers of asthma and allergies in industrialized nations over the last 60 years [46,47]. Between 1980 and 1996, the number of people who have asthma went from 6.5 million to 14.5 million in the United States [48]. China is currently the world’s major consumer and producer of phthalates [49]. China produces around one-fourth of the entire amount of phthalates used worldwide [50,51]. In previous studies, Wang et al. found that the concentration of indoor phthalates in China was higher than in developed countries such as the USA, which indicates a more severe condition in Chinese building environments [50].

SVOCs (PAHs, PAEs, PBDEs, PCBs) are known as endocrine-disrupting chemicals (EDCs). EDCs have a wide range of influences on human health, and it is a universal concern at all stages of human life, from the embryonic development to the elderly, who are consistently exposed to low concentrations of these chemical compounds. It is our objective, therefore, to summarize the known health impacts of SVOCs in the Chinese built environment as established by published literature. This information may be used for the establishment of Chinese standards on indoor SVOC exposure and for enlightening prospective studies.

## 2. Materials and Methods

### 2.1. Literature Searching

We searched all published literature on SVOC exposure and health effects in Chinese building environments between 1980 and 2017. The databases included China National Knowledge Infrastructure in Chinese (CNKI), WANFANG DATA (in Chinese), PubMed, and Web of Science. The search terms were categorized as:

Indoor pollutants: semi-volatile organic compounds, SVOCs, phthalic acid esters, phthalates, PAEs, polycyclic aromatic hydrocarbons, PAHs, benzopyrene, BaP.

Building type: university, college, school, classroom, institutes, office, home, house, dwelling, residence, apartment, kindergarten, daycares.

Health outcomes: Search terms were selected based on the World Health Organization (WHO) and the International Programme for Chemical Safety (IPCS) guidelines. They suggested 122 terms, including allergies, respiratory diseases, and cancer (see Supplementary Materials for more details).

The reference management program EndNote, version X8, Thomson Scientific, Stamford, CT, USA, was used to enter all of the retrieved papers and check for duplication.

### 2.2. Literature Screening

A total of 5477 papers were obtained in the literature search. Papers which provided information on indoor SVOC exposure and their impact on human health were selected; the rest were eliminated. Screening and evaluation of the papers occurred in two steps: First, we determined relevance based on the title and abstract of each article. Second, we read the full texts of the remaining papers. Papers were excluded if (1) studies had not been performed in China; (2) studies had been carried out on animals; (3) in vitro or in vivo studies had been performed on cells; (4) studies showed no data on health outcomes; (5) health risks were estimated with the use of models or equations; and (6) the studies investigated the health effects of pollutants on rivers, soil, vegetables, biomarkers, or ambient air. The screening process yielded 58 papers for further examination by the review panel. Prior to the workshop, the panel studied these 58 publications, and at the workshop, they discussed the papers and created a consensus statement based on the available evidence.

### 2.3. Literature Review

The review panel consisted of 5 people, with backgrounds in Environmental Science, Environmental Engineering, Public Health, and Built Environment. The full texts of the chosen papers were reviewed by two independent review panel members to avoid bias.

Reviewers retrieved data from each article, specifically the type of study, setting/location, investigated population, indoor pollution and health outcomes, potential sources of bias, research results, and conclusions. A primary reviewer verbally summarized each of the 58 papers during the workshop. A second reviewer provided further feedback. The review panel’s broad discussion of each article resulted in its ultimate categorization into one of the following categories:

- Relevant and (partially) conclusive—giving sufficient information regarding exposure to indoor SVOCs and its health outcomes, and relationship between indoor SVOC exposure and its health outcomes.
- Relevant and suggestive (background)—although not conclusive, there is some suggestion that exposure to indoor SVOCs and health outcomes may be associated—or neither.
- Irrelevant—not addressing a topic covered by the review; lacking information on exposure to indoor SVOCs and its health outcomes.

## 3. Results and Discussion

**Figure 1:**
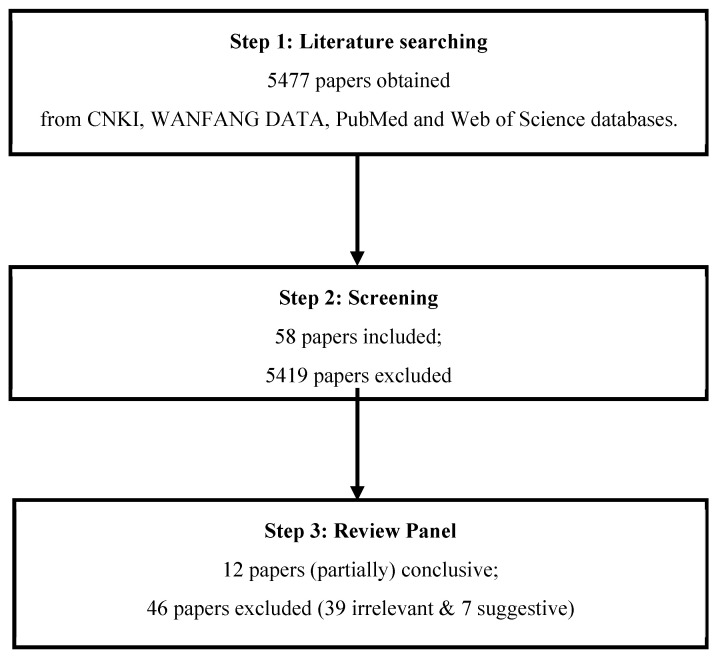
Flowchart of the literature review.

As shown in Figure 1, out of the fifty-eight publications reviewed by the panel, thirty-nine were determined to be irrelevant and seven were deemed to be suggestive (see Supplementary Materials, Table S1), while twelve were deemed to be (partially) conclusive and were used to create the consensus statement.

**Figure 2:**
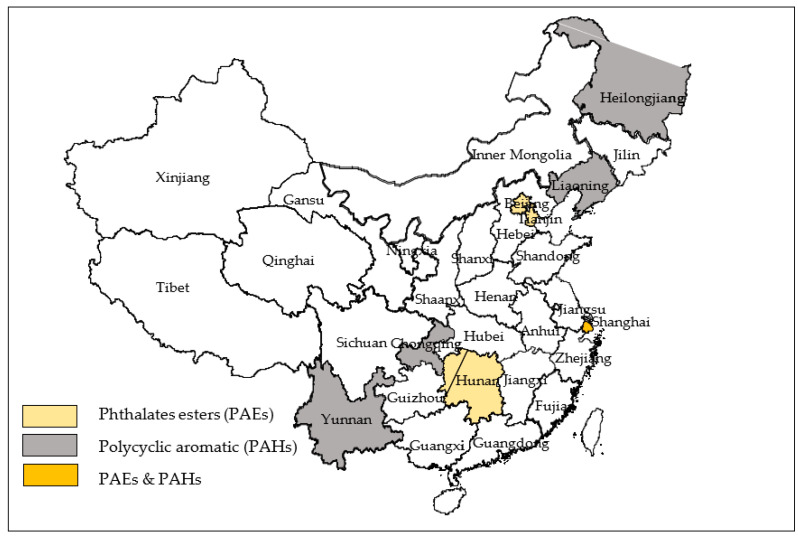
The locations of all studies on health effects of SVOCs in China.

These 12 studies were carried out in eight provinces/municipalities in China, namely, Beijing, Tianjin, Shanghai, Chongqing, Yunnan, Liaoning, Heilongjiang, and Hunan Province. Figure 2 illustrates the location of these investigated areas. None of these studies covered the northwest region of China.

The health effects of PAHs were examined in six papers, while six papers investigated the associations between phthalate exposure and health outcomes. Only one paper investigated the health effects of PBDEs, OCPs, and PCBs [52]. Nie et al. found, compared to healthy children, children diagnosed with asthma had considerably higher concentrations of PBDEs in their indoor fine particulate matter (PM2.5) (41.1 pg/m^3^ v.s. 23.8 pg/m^3^). Indoor exposure to PBDEs may be linked to a risk of developing asthma in children [52].

### 3.1. Polycyclic Aromatic (PAHs) and Health Outcomes

Table 1 presents the findings on PAH exposure and its associations with human health. Out of six studies, four studies focused on PAH exposure and lung cancer [53,54,55,56], one on asthma [52], and one on the ability of population learning and memory [57].

Based on the reviewed studies in Table 1, it can be concluded that higher PAH exposure is extremely likely to be risk factor for lung cancer [53,54,55].

Generally, PAHs are lipophilic substances that simply pass through cell membranes after inhalation via passive diffusion. Following entrance into the lung, PAHs activate phase I metabolic enzymes through both aryl hydrocarbon (AhR)-dependent and -independent routes. The transformation of PAHs into their carcinogenic metabolites is a factor in the etiology of cancer [58]. B[a]P is typically utilized as a marker for overall exposure to carcinogenic PAHs [59]. The average B[a]P content in indoor air in our reviewed studies ranged from 0.01 µg/m^3^ to 6.29 µg/m^3^, which exceeded both the WHO’s and China’s indoor air quality standards [60,61,62,63] (i.e., 0.001 µg/m^3^ for B[a]P (24 h average)), especially in Xuanwei, where the rate of people with lung cancer was higher than in other cities in China. This could be explained by the continued use of solid fuels [55] for heating and cooking [28] by many of the residents. Exposure to indoor smoky coal combustion has been linked to an increased risk of lung cancer [64,65]. Coal is often used in rural places due to its abundance and low cost [66]. Although developing countries like China face more lung cancer caused by fossil fuels, a small number of studies have investigated this issue in developed countries. In Los Angeles, CA, a sample of Caucasian women, who were at risk of developing cancer reported previous exposure to coal/wood stoves and fireplaces at an early age [67]. A study conducted in eastern Europe and Britain demonstrated a 20-40% risk of developing lung cancer among those who used solid fuels in the kitchen, as opposed to those that used it only for heat [68].

Additionally, this review showed the influence of PAH exposure on other outcomes such as asthma and population learning. Nie et al. suggested that some common PAHs combined with indoor PM2.5 might be linked to the risk of developing asthma in children [52]. This is consistent with a comparable study conducted in Moravia, Czech Republic, which linked elevated PAH exposure to intrauterine growth retardation, as well as to asthma [69].

Evidence was also presented for the neuropsychological effects of PAHs following occupational exposure. He investigated coke oven workers in Chongqing, a municipality of China, with a series of neurobehavioral tests, along with urinary 1-hydroxypyrene (a metabolite of PAHs) tests and found that PAH exposure caused a decline in population learning and memory, with increased monoamine neurotransmitters among the workers [57]. A similar trend was observed in a Korean research study conducted by Cho et al., who found that PAH exposure was linked to a decline in memory function and verbal learning in healthy adults [70].

This review showed the primary source of PAHs was solid fuel combustion [53,54,55], such as with coal. Therefore, the health effects of indoor PAH exposure can be decreased by limiting the sources, replacing them with clean, alternative energy, and by providing adequate ventilation. The role of ventilation in decreasing PAH exposure was highlighted in a study that showed that women between the ages of 20 and 40 years old, who did not use the fume hood at all, had a 2.47 times higher likelihood of developing cervical cancer than those who used it all the time [71].

### 3.2. Phthalates and Health Outcomes

Table 2 presents the findings on PAE exposure and its associations with human health. All six existing studies focused on asthma and allergy health outcomes [4,6,72,73,74,75].

The data in Table 2 show that most studies focused on the primary constituents of phthalates in homes such as DEHP, DBP, DEP, and DiBP, which were supposed to be associated with allergies, asthma, and other respiratory diseases. Two studies in Tianjin reported significant positive associations between DEP and DiBP and asthma, DEP and DEHP and dry cough among children, and DBP and rhinitis among younger adults. In the studies conducted in Shanghai, Beijing, and Hunan Province, there was no clear association between phthalate exposure and asthma and allergy. It is possible these studies were limited by their sample size and design (around ten samples in the Beijing study [4], twelve in Shanghai [6] and ten in Hunan [74]). Whether or not these sample sizes have an influence on these studies, the findings do highlight the necessity for improved documentation of phthalate measurements and research design in future investigations of indoor environments and health.

The prevalence of allergies and asthma has increased in developed countries, such as Western Europe, Australia, the USA, and New Zealand [76]. As developing regions, such as China, progress, the prevalence of asthma and allergies has also increased [77]. Over the past 30 years, there has been a significant change in the pollutants released indoors due to the increased use of plastics, synthetic wood products, wall coverings, polymeric floors, and cleaning agents [78]. The trend is apparent, as the standard of living is improving, as is the exposure to phthalates and the burden of diseases. This is consistent with comparable studies conducted in Sweden, which linked elevated eczema and rhinitis rates with BBzP and established the association between asthma and DEHP among children [79], which was subsequently confirmed by Bulgaria’s ALLHOMES study [80]. Bamai et al. also found an association between phthalate and asthma and allergies among Japanese children and adults [81]. The link between phthalate exposure and nasal, airway, ocular, and dermal allergy outcomes was supported by a review study by Bølling et al. [82]. These epidemiological studies from our review and other countries offer evidence of the relationship between asthma and allergy and phthalate exposure.

### 3.3. Research Gaps and Future Considerations

Although China is vast, research on phthalates and their health effects has mostly been conducted on children in Central, South, North, and Eastern China, where the majority of China’s big cities are situated [83]. Most studies related to PAHs and their health outcomes were conducted predominantly on adults who live in Southwest, East, and Northeast China. However, semi-volatile organic compounds affect people (children, adults, and the elderly) all across the country, depending on their living conditions, making nation-wide assessments necessary. Furthermore, despite the fact that this has been extensively studied outside of China, no study in our review sought to investigate the connection between SVOC exposure and the emergence of disorders in pregnant women and their offspring. Overall, our review shows that the number of studies is too limited to definitively link SVOC dosage to health outcomes.

To our knowledge, this paper is one of the first systematic reviews on the health effects of exposure to SVOCs in Chinese buildings. We find that the number of published studies in China is very limited, even though SVOC exposure is an extremely important topic. There is a need to know more about SVOCs to plan for a healthy future. To start, comprehensive studies are needed throughout China to account for rapid urbanization, changes in lifestyle, new indoor building materials, and the burden of new diseases due to modern chemical compounds such as SVOCs. This can serve as a stepping stone for more extensive studies in the future. The authors’ suggestion to the relevant stakeholders would be to develop a Chinese standardized IAQ protocol that assesses SVOC levels, along with other toxins, and put forward policies in line with national and international requirements.

## 4. Conclusions

The objective of this review was to present a thorough summary of the available information on the impact of SVOC exposure on human health in Chinese built environments. In doing so, this review concluded that asthma and allergies may be linked to phthalate exposure, and lung cancer may be linked to PAH exposure. In order to improve indoor environments, along with safeguarding human health, SVOC levels have to be drastically reduced.

## Figures and Tables

**Table 1:** Polycyclic aromatic (PAH) exposure and its associations with health outcomes.

References	Health Outcomes	Types of Study	Settings/Locations	Investigated Population	Indoor Pollutants	Findings
Wang and Dai (1989) [53]	Lung cancer	Case–control	Homes in Harbin, Heilongjiang Province	55 females with lung adenocarcinoma and 55 healthy females aged 30–69 (±5) years old	B[a]P	The highest risk variables for lung cancer were a high coal consumption index (odds ratio (OR): 10.59) and indoor smog pollution (OR: 15.19). The daily amounts of total suspended particulate (TSP) and B[a]P in residents’ bedrooms were 4.4 and 26.7 times higher indoors than outdoors in the winter. The indoor daily average concentration of B[a]P was 0.0106–0.0598 μg/m^3^.
China Preventive Medical Center (1984) [54]	Lung cancer	Case–control	Homes in Xuanwei County, Yunnan Province	People in areas with high/low incidence of lung cancer	B[a]P	The indoor concentrations of B[a]P in lung cancer high-incidence areas (6.29 µg/m^3^) were significantly higher (more than ten times) than in low-incidence areas (0.45 µg/m^3^).
Lan et al. (2008) [55]	Lung cancer	Case–control	Homes in Xuanwei, Yunnan Province	498 farmers with lung cancer as a case group and 498 farmers as a control group	Smoky coal subtype as an indicator of BaP exposure	When compared to the use of smokeless coal or wood (OR = 7.7, 95% confidence interval (CI) = 4.5–13.3), the influence of smokey coal (with subtypes of Laibin and Longtan) on lung cancer was significantly stronger (24.8 (95% CI: 12.4–49.6)).
Hoshuyama et al. (2006) [56]	Lung cancer	Cohort	Iron and steel manufactories in Anshan, Liaoning Province	121,846 workers in iron and steel factories	PAHs	PAH exposure was a main risk factor for lung cancer in exposed workers with standardized rate ratios (SRR) of 159 (95% Confidence Interval: 115–219).
Nie et al. (2016) [52]	Asthma	Case–control	Homes in Shanghai	29 children (3-6 years old) with diagnosed asthma as case group and 31 healthy children as control group	PAHs	In comparison to the control group (0.0442 µg/m^3^), the case group with asthma had considerably higher concentrations of PAHs in their indoor PM2.5 (0.0572 µg/m^3^).
He (2010) [57]	Ability of population learning and memory	Cohort study	Factories in Chongqing	100 male workers in cock plant of a steel company and 100 male workers of oxygen installation	B[a]P	The occupational exposure of B[a]P for coke oven workers was 0.27–2.47 ug/m^3^, and it was significantly higher than that for oxygen installation workers (0.025 ug/m^3^). Tension–Anxiety(T) and Fatigue–Inertia(F) of the Profile of Mood States (POM S) in coke oven employees were substantially greater than in controls (*p* < 0.05). Compared to the coke oven workers, the scores of the oxygen installation workers were higher in the total digital span, average simple reaction time, the forward digital span, first right dotting, digit symbol, mean right dotting, and mean total dotting, and the differences were statistically significant (*p* < 0.05).

**Table 2:** Phthalate ester (PAE) exposure and its associations with health outcomes.

References	Health Outcomes	Types of Study	Settings/Locations	Investigated Population	Indoor Pollutants	Findings
Hu (2017) [74]	Allergic diseases	Case–control	10 homes in Changsha, Hunan Province	10 children aged 10–12 years old	DEHP and DBP	The concentrations of DEHP (891 ug/g dust) and DBP (263 ug/g) in allergic children’s homes were higher than those in homes with healthy children (DEHP: 755 ug/g, DBP 200 ug/g). However, the difference was not significant (*p* > 0.05).
Zhang (2016) [73]	Asthma and allergy	A nested case–control	Home in Tianjin municipal and Cangzhou city	410 children aged 0–8 years old	DEP, DiBP, DnBP, DEHP, BBzP, DiNP	The median concentrations of DEP, DiBP, BBzP, DnBP, DiNP, and DEHP were 0.31 μg/g, 16.38 μg/g, 0.11 μg/g, 42.6 μg/g, 0.28 μg/g, and 127.11 μg/g, respectively, in investigated homes. The adjusted odds ratios (AOR) for children with diagnosed asthma were 2.08 and 2.48, respectively, when the concentrations of DEP and DiBP in dust in urban residential buildings were above 0.33 µg/g and 16.38 µg/g. Children’s dry cough was linked to increased DEP and/or DEHP concentrations in rural areas.
Fan et al., (2017) [4]	Respiratory and allergic symptoms	Case–control	Homes in Beijing	Six children (8–12 years old) diagnosed with allergic and respiratory symptoms as case group (group A) and four healthy children as control group (group B)	DEHP, DBP, DBA, DOA, and DEP	The mean concentrations of SVOCs in living rooms and bedrooms of thecontrol group (1590 µg/g and 2347.8 µg/g) were higher than the case group (1347.5 µg/g and 1754 µg/g) in winter. In summer, the mean concentrations of SVOCs in living rooms and bedrooms of the case group were higher than the control group (except one home, and two homes were detected with no SVOCs).
Zhang et al. (2016) [6]	Allergy	Case–control	Home in Shanghai	Seven children (9–10 years old) diagnosed with allergic symptoms as case group and five healthy children as control group	DEP, DBP, DEHP, BHT, DBA, DOA, TBP, TCEP, TPP	The concentrations of DEHP and DBP were not substantially different between healthy and allergic children.
Sun (2008) [72]	Allergies	A nested case–control	Dormitories at Tianjin University	209 students diagnosed with allergic symptoms as case group and 227 healthy students as control group	DEP, DiBP, DBP, BBzP, DEHP, and DiNP	The average indoor concentrations of DEP, DiBP, DBP, BBzP, DEHP, and DiNP were 21.38, 23.09, 24.90, 21.75, 48.54, and 31.95 μg/g. The adjusted odds ratio (AOR) for diagnosed rhinitis was 5.03 (1.32–19.14) in the dust of rooms with a concentration above 24.9 μg/g.
Zhang (2017) [75]	Eczema, allergic rhinitis	Case–control	146 Dormitories at Beijing University of Civil Enginering and Architecture. 74 were case rooms and 72 were control rooms	College students lived in these dorm rooms	DBP, DCHP, DiBP, and DEHP	The concentrations of DBP (43–60 μg/g), DCHP (119–155 μg/g), and DEHP (149–181 μg/g) in the dust of allergic students’ dormitories were similar to those in healthy students’ dormitories (DBP 20 μg/g, DCHP 126 μg/g, and DEHP 152 μg/g, respectively). The difference was not significant (*p* > 0.05).

## Data Availability

The data are available from the corresponding authors upon reasonable request.

## Supplementary Materials

The following supporting information can be downloaded at: https://www.mdpi.com/article/10.3390/ijerph20010678/s1, Literature Searching Items for Health Outcomes; Table S1: Summary of Suggestive Studies on the Exposure to Phthalate esters (PAEs) and Polycyclic aromatic (PAHs) and health outcomes. References [5,28,71,84,85,86,87] are cited in the Supplementary Materials.

## Abbreviations

AOR: adjusted odds ratios; B[a]P: benzo[a]pyrene; BBzP: Butyl benzyl phthalate; BHT: butylated hydroxytoluene; CI: confidence interval; DBA: dibutyl adipate; DBP: Dibutyl phthalate; DCHP: Dicyclohexyl phthalate; DEP: Diethyl phthalate; DEHP: Di (2-ethylhexyl) phthalate; DiBP: Diisobutyl phthalate; DiNP: Diisononyl phthalate; DOA: di(2-ethylhexyl) adipate; OCPs: Organochlorine Pesticides; OR: odds ratios; PAHs: Polycyclic aromatic hydrocarbons; PAEs; Phthalate esters; PBDEs: Polybrominated diphenyl ethers; PCBs: Polychlorinated biophenyls; SRRs: standardized rate ratios; SVOCs: semi- volatile organic compounds; TBP: tributyl phosphate; TCEP: tris(2-chloroethyl) phosphate; TPP: triphenyl phosphate; TSP: total suspended particulate.
