# Supplementary material for: Health Effects of Exposure to Indoor Semi-Volatile Organic Compounds in Chinese Building Environment: A Systematic Review
Source: Int J Environ Res Public Health. 2022 Dec 30;20(1):678. doi: 10.3390/ijerph20010678 (PMC9819628; doi:10.3390/ijerph20010678)
Supplement: Supplementary file 1 [file ijerph-20-00678-s001.zip › ijerph-2088328-supplementary.pdf]

Supplementary materials

Health effects of exposure to indoor semi-volatile organic compounds in Chinese building environment: a systematic review

Yeganeh Ataei [1], Yuexia Sun [1,*], Wei Liu [1], Agnes S. Ellie [1], Hui Dong [2], Umme Marium Ahmad [1]

1 Tianjin Key Laboratory of Indoor Air Environmental Quality Control, School of Environmental Science and Engineering, Tianjin University, Tianjin 300350, China;

2 Centre for Environmental Policy, Imperial College London, South Kensington, London, SW7 2AZ, United Kingdom;

3

* Correspondence: yuexiasun@tju.edu.cn

Contents

Literature Searching Items for Health Outcomes

Table S1. Summary of Suggestive Studies on the Exposure to Phthalates esters (PAEs) and Polycyclic aromatic (PAHs) and Health Outcomes

Literature Searching Items for Health Outcomes

Tumour, tumor, malignant lymphoma, multiple myeloma, non-Hodgkin lymphoma, lung tumor, alveolar tumor, hepatocellular carcinoma, hepatoma, liver tumor, mouth neoplasm, melanoma, oral carcinoma, lymphatic sarcoma, lymphoma, skin neoplasin, dermatoma, esophageal tumor, gastroenteric tumor, reticular granuloma, bronchiolar adenoma, stomach neoplasm, bronchial neoplasms, leukemia, myeloid leukemia, lymphatic leukemia, myelogenous leukemia, macrocythemia, macrocytosis, anemia, arteriosclerosis, leukopenia, pancytopenia, cerebrovascular diseases, ischemic heart disease, cardiovascular diseases, acidosis, cataract, eye irritation, conjunctivitis, nerve system disease, epilepsy, Alzheimer's disease, hyperactivity, stroke, polyneuropathy, cerebral infaction, cerebral thrombosis, permanent encephalopathy, neurodegenerative disease, Parkinson's disease, specific neuropsychiatric disorder, depressive disorder, depression, autism, infantile autism, chronic non-communicable diseases, headache, cold, allergy, contact dermatitis, irritability, chloracne, skin irritation , eczema , dermatitis, oral ulcer, canker sore, anabrosis, respiratory disease, throat irritation, nasal irritation, nasal stimulation, rhinitis, wheeze, pulmonary edema, pulmonary impairment, pulmonary congestion, pneumonia, respiratory tract infection, allergic rhinitis, asthma, cough, influenza, chronic obstructive pulmonary disease, sore throat, airway obstruction, bronchial spasm, bronchial asthma, bronchoedema, bronchial obstruction, bronchitis, effusive pulmonary edema, metabolic syndrome, recurrent urinary calculi, obesity, kidney dysfunction, liver dysfunction, hypertension, hepatomegaly, connective tissue disease, circulation system disease, gastrointestinal injury, liver injury, proliferative nephritis, glomerulonephritis, interstitial nephritis, diabetes mellitus, diabetes, low birth weight, birth defects, preterm birth, premature birth, cancer, rhino carcinoma, nasopharyngeal carcinoma, nasal cavity carcinoma, colorectal cancer, lung cancer, oral cancer, ovarian cancer, bladder cancer, cerebral cancer, skin cancer, forestomach cancer, trachea carcinoma ,breast cancer, ureter cancer, kidney cancer, gastric cancer, stomach cancer, small cell carcinoma, hypo pharyngeal cancer, pancreatic cancer, throat cancer, metro carcinoma, bronchogenic carcinoma, uterine cancer.

Table S1: Summary of Suggestive Studies on the Exposure to Phthalates esters (PAEs) and Polycyclic aromatic (PAHs) and Health Outcomes

References	Health outcomes	Types of study	Settings/Locations	Investigated population	Indoor pollutants	Findings
Kim et al. (2014) [84]	Lung cancer	Case-control	Homes in Xuanwei, Yunnan Province	260 men (farmers aged 18-85 years) diagnosed with lung cancer as case group, 260 randomly sampling of normal male as control group	Smoky coal, tobacco smoking (PAH)	Smoky coal usage has a significant association with increased risk of lung cancer. In comparison, the association of increased risk of lung cancer due to cigarette smoking is non-significant.<br>As the quantity of smoky coal used increases, the odds ratio (OR) of developing lung cancer due to cigarette smoking decreases (>0-3 tons: OR: 1.09; 95% confidence interval (CI): 1.03-1.17; >3 tons: OR: 0.99; 95% CI: 0.95-1.03). For Lai Bin and Long Tan users, the OR or risk due to cigarette smoking drops further, even at low levels of usage (>0-3 tons: OR: 1.02; 95% CI:0.91-1.14; >3 tons: OR: 0.94; 95% CI: 0.97-1.03).
Chuang et al. (1992) [85]	Lung cancer	Cross-sectional	Homes in Xuanwei, Yunnan Province	Three (3) communes (Cheng Guan, Re Shui, Lai Bin) involving those with high lung cancer	PAH	An important contributing factor to the elevated lung cancer mortality rates in Xuan Wei was the indoor burning of smokey coal under fabricated settings. In addition, smoky coal samples had greater indoor PAH concentrations than wood and smokeless coal samples, respectively.

Lan et al. (1993) [86]	Lung cancer	Case-control	Homes in Xuanwei, Yunnan Province	139 female diagnosed with primary lung cancer as cases and 139 health females as controls	Smoky coal subtype (PAH)	Laibin (smoky coal) use was linked to an elevated risk of developing lung cancer. (odds ratio=9.89, 95% confidence interval= 3.95-24.75).
Wu et al. (2004) [71]	Cervical intraepithelial neoplasm	A nested case-control	Homes in Chia-Yi County, Taiwan	100 women diagnosed with cervical intraepithelial neoplasm above of 19-year-old as cases and 197 healthy women as controls	Exposure to cooking oil fumes (such as PAH)	Women from the ages of 20 to 40, who were in the kitchen at least once a week without using the fume hood presented an increased chance of being diagnosed with cervical intraepithelial neoplasm (adjusted odds ratios=2.29, 95% (CI) = 1.08–4.87). Women who did not use the fume hood at all had a 2.47 times higher likelihood of cervical cancer than those who used it all the time. Women who reported to have used a fume hood all the time between the ages of 20 to 40 and continued to do so after 40 years of age had significantly lower odds of developing cervical cancer in their senior years
Li et al. (2011) [28]	Risk of neural tube defects (NTD)	Case-control	Homes in Shanxi Province	610 neural tube defects (including aborted fetuses, prenatally diagnosed, stillborn infants and, live born infants) as cases and 837 healthy infants as controls	Indoor air pollution from coal combustion (PAHs)	NTD risk was associated with both cooking (OR = 1.5, 95% CI: 1.1, 2.1) and residential heating (OR = 1.7, 95% CI: 1.1, 2.4).
Wei et al. (2018) [87]	Gross motor functions	Cohort	Homes in Taiwan	15,310 singleton infants which were	Household incense	The occasional or persistent indoor incense burning were linked to delays in children's reaching

				six (6) and, eighteen months	burning (such as PAH)	developmental milestones such as walking with support among the occasional incense burning: OR=1.26, 95% (CI): 1.08 to 1.47, persistent incense burning: OR=1.44, 95% CI: 1.22 to 1.69 and, walking steadily due to occasional incense burning: OR=1.14, 95% CI: 0.98 to 1.32, persistent incense burning: OR=1.24, 95% CI: 1.06 to 1.45.
Hu et al. (2017) [5]	Allergy	Case-control	Homes in Harbin, Dalian, Beijing, Shanghai, Wuhan, Changsha cities	60 students with 4th- and 5th-grade school (ten children from each city). Children diagnosed with allergy symptoms as cases and healthy students as controls	DBP and DEHP	The exceeding concentrations of DBP and DEHP than 1000 μg/g may increase the allergy in children.
